# Tweeting and Eating: The Effect of Links and Likes on Food-Hypersensitive Consumers’ Perceptions of Tweets

**DOI:** 10.3389/fpubh.2018.00118

**Published:** 2018-04-23

**Authors:** Richard J. T. Hamshaw, Julie Barnett, Jane S. Lucas

**Affiliations:** ^1^Department of Psychology, University of Bath, Bath, United Kingdom; ^2^Clinical and Experimental Sciences, Faculty of Medicine, University of Southampton, Southampton, United Kingdom

**Keywords:** food hypersensitivity, food allergy, food intolerance, social media, Twitter, Uses and Gratifications, Elaboration Likelihood Model

## Abstract

Moving on from literature that focuses on how consumers use social media and the benefits of organizations utilizing platforms for health and risk communication, this study explores how specific characteristics of tweets affect the way in which they are perceived. An online survey with 251 participants with self-reported food hypersensitivity (FH) took part in an online experiment to consider the impact of tweet characteristics on perceptions of source credibility, message credibility, persuasiveness, and intention to act upon the presented information. Positioning the research hypotheses within the framework of the Elaboration Likelihood Model and Uses and Gratifications Theory, the study explored motivations for using social media and tested the impact of the affordances of Twitter—(1) the inclusion of links and (2) the number of social validation indicators (likes and retweets). Having links accompanying tweets significantly increased ratings of the tweets’ message credibility, as well as persuasiveness of their content. Socially validated tweets had no effect on these same variables. Parents of FH children were found to utilize social media for social reasons more than hypersensitive adults; concern level surrounding a reaction did not appear to alter the level of use. Links were considered valuable in obtaining social media users to attend to useful or essential food health and risk information. Future research in this area can usefully consider the nature and the effects of social validation in relation to other social media platforms and with other groups.

## Introduction

As the structure and function of online media has developed to enable more active citizen involvement, understanding why we use and respond to social media is of increasing interest to scholars exploring online behavior (1). Access to social media platforms and their two-directional communication affordances means Internet users can readily connect with others and share information. One area in which social media is receiving increasing attention is around managing food risk—both in relation to the activities of those that seek to manage the risks to which consumers might be exposed (2–5) and of the ways in which consumers interact with social media as part of their information-seeking activities (5). Building on this, the more particular focus here is upon how judgments about the credibility and persuasiveness of social media information and intentions to act upon it are affected by structural elements of the social media communication. We focus on Twitter as a widely used and researched social media platform that (6) in a context where social media is increasingly used by risk communicators around food issues (7). We address this with a group that has particular reason to consider the veracity and provenance of information about food—food-hypersensitive (FH) consumers that are seeking to avoid food that contains particular allergens. Twitter is a useful tool for this community for gathering or sharing important and useful information as well as seeking social support (8).

The introduction will unfold as follows. We first seek to characterize the research that has addressed how consumers use social media information in relation to food and introduce the social media platform—Twitter—that is the focus of this study. Having outlined how information on social media may have particular salience for those with FH, we introduce the theoretical perspectives that frame the study: Uses And Gratifications Theory (UGT) and the Elaboration Likelihood Model (ELM), before outlining the exact focus on the present study and the hypotheses that will be addressed.

### Social Media and Food-Related Information

There is a small but growing body of evidence about the way in which, and the reasons why, people use social media in relation to food. Consumers regularly use social media to both seek and share information about food, illustrating passive and active behavioral approaches. In a more passive sense, consumers might seek information about products, recipes, diets, healthy eating, and risks (9–11). Alternatively, users may actively share food information themselves (12) or seek support/advice from their online peers (13, 14). This active collective participation on social media is highly significant; users take notice of the information posted by other users and not just authorities on the topic. The origin, credibility, and content of social media posts and related comments are features users routinely consider when making judgments about food safety information (15). Other research has analyzed consumer engagement with social media to show patterns of coping with food-risk incidents, such as information-seeking around appropriate food choice or handling (16, 17) as well as the way in which expert presentations of the underlying food science can be dismissed, discounted, or contested when the information is incongruent with their opinion (18).

Social media has quickly become an expansive resource of information and can give users access to some of the most engaged members of the public (5, 6). Consequently, social media has a role in public discourse around risks and concerns, has an information-providing function, and signals a decreased dependence on traditional media outlets (19). The increase in information sources available on social media may amplify consumer perceptions of risk and uncertainty, making this an important area of research (20). Stakeholders no longer need to go through traditional media channels; they can report an issue exactly when and how they wish (4). Increasingly, such bodies utilize this advantageous feature of social media for food-risk communication as well as health and safety campaigns (5, 21–23). Platforms allow for the rapid distribution of information and can assist in managing public reactions toward risk events, as well as encourage appropriate behavior, calm, educate, and increase awareness (4, 23, 24). Organizations also recognize that the information consumers themselves post on social media can provide important intelligence that can inform their risk management strategies (25) or food choice decisions relating to consumer perceptions of health and well-being (12). Thus, food organizations are pushing information out to consumers and may also seek to learn from the information that consumers are posting (3, 23).

Twitter is a social media platform that is increasingly important in food-risk communication. Although less popular than Facebook, it is widely used with 17.1 million UK users in 2018 (26). As a real-time news-style platform, information can be rapidly disseminated, publicly challenged, and can spread and become established through the process of retweeting (4). Information on Twitter takes the form of comments which can contain the names of other Twitter users, hashtags (#)—which function to organize themes across tweets—and links to other media sites (URLs). Each tweet is associated with information as to the number of times that it has been shared (retweeted) and liked (27). The availability of this information has served to focus research interest (6). Indeed, Tufekci (28) contends that the clean and simple structure of Twitter enables it to serve as a “model organism” that “facilitates progress in basic questions underlying the entire field” (p. 506). This simplicity has allowed for the creation of stimuli to explore perceptions of Twitter information within an experimental study design.

Twitter has become a key communication tool for organizations seeking to manage risk (2, 23). This is not only because it provides a channel to send information out as part of a public health campaign, for example, but also due to the potential insights provided through content analysis of tweets (12, 16), sentiment analysis of Twitter data (17), linking Twitter data with other types of data such as demographics to provide information about users themselves (29), or overlaying tweet content with geolocation data (30). The UK Food Standards Agency has recently analyzed Twitter data to help detect outbreaks of Norovirus in order to inform the timing and location of interventions (31). In line with the work by Gaspar et al. (16, 17) who look at what tweets reveal about coping patterns with a food contamination incident, the current study also considers individual patterns of Twitter use. Our focus is upon understanding how the functionality of Twitter can influence public perceptions of a message independently of the content of tweets. We explore the activities of users and the affordances of the Twitter platform (32) in relation to a group of users who have a particular motivation to avoid and to manage risk in relation to food consumption: those seeking to avoid allergens in their food.

### Food Hypersensitivity

Food hypersensitivity refers to individuals who suffer reproducible negative symptoms whenever they eat a particular food and denotes both food allergy and non-allergic FH [e.g., food intolerance and celiac disease (33)]. Living with FHs involves constant risk assessments surrounding the foods one consumes. This is especially the case when eating outside the home (34). Those with food intolerance wish to avoid repeatable adverse reactions to foods such as bloating, constipation, vomiting, and diarrhea. Food-allergic individuals, in more severe cases, need to avoid allergen consumption that could lead to anaphylaxis (associated with breathing difficulties, sudden drop in blood pressure, and potential death).

The role of social media in providing information or social support for people with FHs has received little empirical attention. Given that there is no cure for FH and the prevention of a reaction by avoiding consumption of the offending food allergen is vital, it is not surprising that social media provides information (e.g., product alerts) and sources of support through forums, discussion groups, blogs, and microblogs (8). Social media has also increasingly become a platform for industry, support groups, and those with regulatory responsibilities to circulate information relating to FH. The Food Standards Agency (@foodgov) routinely tweet allergen alerts and product recall information relevant to allergens.

Recent research has alluded to ways in which FH consumers utilize the Internet to gather information about allergens before eating out (35). Several strategies were employed, including menu-checking *via* websites, using search engines to check if specific dishes usually contain an allergen, as well as using QR-code scanning to check for specific ingredients *via* links. This is particularly significant insofar as consumers with FH had a clear preference for written rather than oral information. This sense of reluctance toward asking staff for information appears to manifest from feelings of embarrassment and a reluctance to be seen as making a fuss or drawing unwanted attention to themselves (35).

Having noted the increasing ubiquity of social media for communicating food risk and the particular salience of this for those that are seeking to avoid allergens, we introduce two theoretical frameworks that are used to (1) situate our consideration of how social media is used by FH individuals (UGT) and (2) how the affordances of Twitter might shape responses to information encountered (the ELM).

### Need Satisfaction Through Social Media

There has long been a focus on the gratifications of media use (36). UGT is “a psychological perspective that examines how individuals use mass media, on the assumption that individuals select media and content to fulfill felt needs and wants” (37) that more recently has been applied to use of the Internet (38, 39) and to social media (40–42). Research has identified a range of factors motivating Internet use such as information-seeking, entertainment, relaxation, and passing the time (38–40). In terms of social media, motivations similarly fall broadly into the realms of information-seeking, passing time, and entertainment, but with greater emphasis on news sharing, social interaction, keeping in touch, and surveying what others are doing (40–44). Thriving groups of specific health-concerned users interact on social media and make use of such platforms for sharing useful information and emotional support [e.g., people living with cystic fibrosis—(45); diabetic individuals on Facebook—(46), and FH individuals on Twitter—(8)]. For people caring for loved ones, social networking platforms can be a good source of reassurance (47).

Research has suggested that the use of Twitter echoes complex purposes relating to information and social engagement such as sharing information to gain attention (48), building networks and engaging with other users (49) as well as distributing and discussing news (50). UGT has recently been used to address questions relating to Twitter (51, 52). Twitter users may gain a range of gratifications related to information and social networking (51). Some research has asserted that the most important Twitter motivation relates to the need for social connection (53), and others have suggested that Twitter is mostly used as an information source, rather than to satisfy social needs (54). However, the research in this area thus far has suggested that the provision of information and of social support may be primary gratifications that using Twitter (as well as other social media platforms) can provide. When we consider the particular situation of consumers with FH, previous research indicates that parents of FH children face particular concerns and challenges around their responsibilities for the care and safety of their child (55), which may manifest as stress, anxiety, and depression (56). Consumers concerned about FH utilize online resources to help avoid consuming allergens they or their child react to (8, 35). Both FH adults and parents access social support *via* social media. However, parents have a unique set of concerns and seek the expertise of other FH parents, in part to help with the responsibility they have in managing their child’s reaction to consuming allergens—which in the case of an allergy may be life-threatening (57). Thus, we hypothesize that due to the greater level of responsibility and challenges FH parents face, they will be more motivated to use Twitter for information and social support:

*H1: Parents caring for a FH child will be more motivated to use social media for information and for social support compared to FH adults*.
  Consumers with FH will vary in the severity of their reactions—following a classification system derived in relation to peanut allergy, reactions can be classified as mild, moderate, and severe (58); the salience of more recent and severe reactions that may be life-threatening nature is more likely to be associated with a greater concern. We would therefore hypothesize that seeking social support and information relevant to past reactions or avoiding future reactions is likely to be more pronounced in these individuals:
*H2: Individuals with high reaction salience will be more motivated to use social media for information and social reasons than those with low reaction salience*.

### The Effects of Tweet Characteristics on Credibility, Persuasion, and Intention

Twitter provides information about the extent to which any particular post (tweet) is socially validated—that is, the extent to which it has been attended to by others either by them retweeting or liking it (27, 59). This is not to say that the act of liking or retweeting indicates agreement with the content, simply that to the viewer these metrics designate it as having been of public interest—or not. Tweets may also contain links to other external sites (*via* a URL). The presence of a link may too function to validate the sense that the views being expressed are not simply those of one individual but are supported by material located elsewhere online (60). The question then arises as to how, if at all, the affordances of links, retweets, and likes affect the assessments of those viewing the tweets. Relevant assessments might include the credibility of both tweet and tweeter, how persuasive the content or source is, and (if relevant) the intention that it might inform future actions.

Faced with a vast array of Internet information, questions regarding how people perceive and assess the credibility of the information they encounter have become particularly salient (40, 61). Given the relative ease with which content can be published and altered online, often coupled with the lack of information verification systems, it is important, albeit difficult, for citizens to evaluate the quality and potential inaccuracies of online information (62, 63). These platforms act as key information networks for individuals who consider being well informed as important (5); inaccurate information relating to life-threatening conditions, such as food allergy, could have serious consequences for consumers (8).

The tenets of the ELM are relevant to considering assessments of credibility and persuasiveness of a message/source (64). The ELM considers two routes of persuasion: (1) the central route, which persuades people who carefully consider a range of information contained within the source message, and (2) the peripheral route, which sees cues such as subjective impressions and surrounding/contextual information persuading individuals who lack motivation or ability to consider a source’s finer details (64). Recent research has considered the relative effects of these routes in social media (65), suggesting that the popularity of a post affected the perceived persuasiveness of the message although this was attributed to both central and peripheral processing. Focusing more on the potential peripheral nature of like/retweet information, Waddell (66) tested if high or low like/retweet levels moderated the effect of credibility and issue importance from online comments. This was not found to be the case; rather suggesting that such features are hard to process (67). Furthermore, inferences about the credibility of a source of health information may be based on perceptions of how professional or official a website design is (68). In another example of how peripheral information can be used to infer credibility, Cheever and Rokkum (69) highlighted how design and testimonials or comments from other web users are often employed to assess the credibility of materials above more formal verifications about the information (e.g., affiliations). Indications of the involvement or approval of others may also act as descriptive norms (70), indicating how common a particular behavior or a view is in a group that one belongs to or identifies with (71). Tweets relevant to those with FH are likely to make a FH identity salient (72), and thus indications of being validated by others to whom it is relevant may function as cues to being persuasive and credible and as encouraging intentions to relevant allergy management actions.

Research on FH certainly highlights the everyday rules of thumb and use of peripheral cues, by, for example, forming judgments of whether allergen management in an eating-out venue will be good on the basis of factors such as labeling and perceived cleanliness of the establishment (55, 73). For those who seek to communicate about allergens on Twitter, it will be useful to understand what peripheral cues might shape reactions to their messages.

In summary then, we would propose that the information embedded in tweets both the links to other information and the numbers of retweets and likes that the post has attracted (i.e., the level of social validation) will affect assessments of the credibility and persuasiveness of the message and intentions to take relevant action:

*H3: Higher levels of likes and retweets from other users on tweets will positively influence ratings of source and message credibility, persuasiveness, and intention*.
*H4: The presence of a link as additional information following a tweet will positively affect message and source credibility, persuasiveness, and intention*.

## Materials and Methods

### Design

A two-by-two between subjects quasi-experimental design was used to assess the impact of adult/parent status and high/low reaction salience on the importance of information-seeking and social-support motivations. A further two-by-two between subjects experimental design was used to assess the impact of tweets with high/low social validation indicators and inclusion of a link/no link. There were four outcome variables: perceived message credibility, source credibility, persuasiveness, and intention.

### Participants

Respondents were primarily recruited from a contact list of FH individuals (or parents of FH children) who had taken part in a related study by Barnett et al. (74), had indicated that they were social media users, and agreed to be re-contacted for subsequent projects by the research team (ethical approval reference number: 16-146). Additional respondents who met the same criteria were also sought through advertisements on the member websites and social media accounts of Celiac UK and Allergy UK. In total, there were 251 questionnaire respondents. Full ethical approval for this research project was granted by the Department of Psychology’s ethics committee at the University of Bath (reference number: 16-275). Table 1 outlines the study population demographics; Table 2 summarizes the characteristics of participant FHs.

**Table 1:** Demographics characteristics of questionnaire sample.

(*N* = 251)	*N* (%)
**Gender**	
Female	228 (90.8)
Male	21 (8.4)
Prefer not to answer	2 (0.8)
**Age category**	
18–24	14 (5.6)
25–34	62 (24.7)
35–44	92 (36.7)
45–54	47 (18.7)
55+	36 (14.3)
**Location**	
UK resident	245 (97.6)
Non-UK resident	6 (2.4)
Adults	155 (61.8)
Parents	96 (38.2)
**Twitter familiarity**^^a^^	
Twitter users	101 (40.2)
Not Twitter users	150 (59.8)

*^a^Twitter use in relation to food hypersensitivity*.

**Table 2:** Characteristics of participant food hypersensitivities.

(*N* = 251)	*N* (%)
**Food sensitivity category**	
Allergy	76 (30.3)
Intolerance	174 (69.3)
Unsure	1 (0.4)
**Allergens causing sensitivity^a^**	
Gluten	149 (59.36)
Cow’s milk	93 (37.05)
Peanuts	71 (28.30)
Egg	54 (21.51)
Other nuts	52 (20.72)
Soya	36 (14.34)
Sesame	20 (7.97)
Fish	11 (4.38)
Crustaceans	8 (3.19)
Mollusks	8 (3.19)
Sulfur dioxide	7 (2.79)
Mustard	6 (2.39)
Lupine	6 (2.39)
Celery	4 (1.59)
Other(s)	50 (19.92)
**Diagnosis type**	
Formal medical diagnosis	218 (86.85)
Alternative diagnosis	8 (3.19)
Self-diagnosis	10 (3.98)
Other	15 (5.98)
**Speed of reaction**	
Immediately	89 (35.46)
Within 1 h, but not immediately	62 (24.70)
1–24 h later	82 (32.67)
After 24 h	18 (7.17)
**Reaction salience**	
High concern	107 (42.6)
Low concern	144 (57.4)

*^a^Can be more than one causative allergen*.

### Materials

The online questionnaire survey was hosted *via* the Qualtrics survey platform.^1^ Initial questions related to demographic and FH information, as well as typical use of social media platforms and those used specifically in relation to FH. In assessing reasons *why* social media sites were potentially utilized for reasons relating to FH, an adapted version of a Uses and Gratifications Social Media measure was employed, taking account of elements relating to information-seeking, social connection, and entertainment gratifications (40). The scale wording was adjusted to fit more appropriately to the FH focus, and thus a factor analysis was conducted to confirm the constitution of the scales.

**Figure 1:**
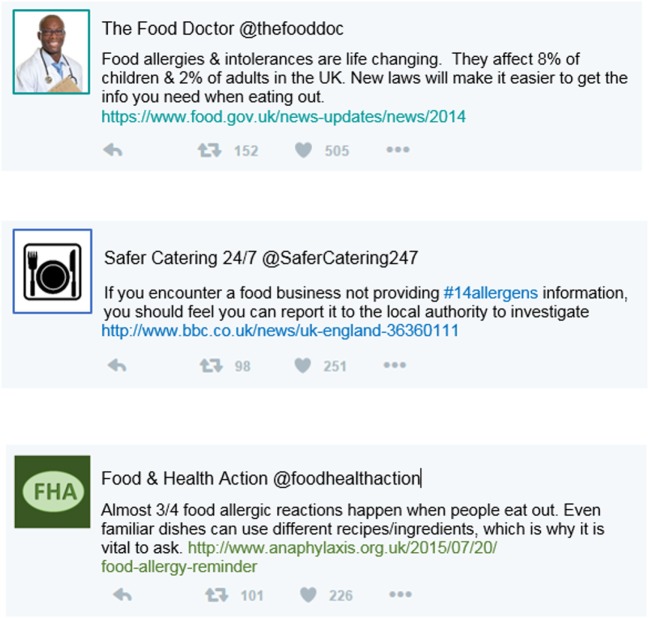
Sample Twitter feed stimuli used for condition 1, showing high retweets and likes, and links included.

**Figure 2:**
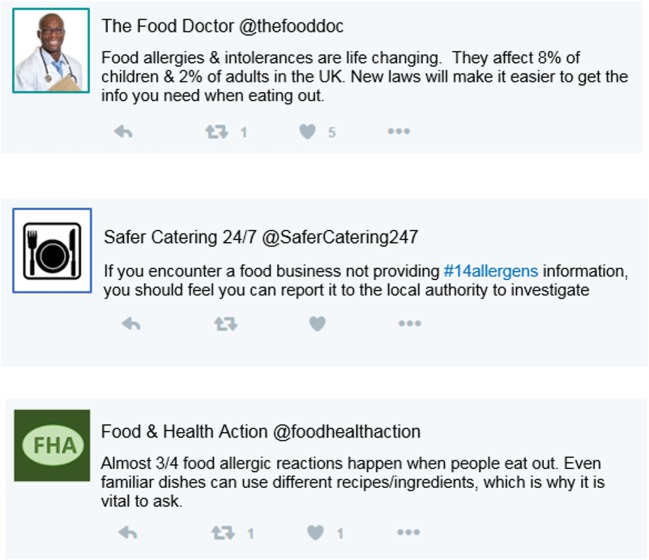
Sample Twitter feed stimuli used for condition 4, showing low retweets and likes, and no link.

Two independent variables (IVs) were experimentally manipulated. Sample Twitter feeds were used to embed manipulations associated with the ELM. High and low levels of social validation (through likes and retweets) established peripheral information cues, high levels were shown with between 98 and 152 retweets and between 226 and 505 likes (Figure 1), and low levels were shown with maximum one retweet and five likes (Figure 2). The inclusion or exclusion of links established central cues.

Manipulation checks were conducted in order to assess if participants had interpreted the IVs as intended. To check for perceptions of social validation, participants were asked to what extent they felt that information from the tweets was appreciated by and shared between social media users with ratings from 1 “not at all” to 5 “extremely.” To check for the perception of link inclusion, the second check asked to what extent participants felt tweets made use of links to other websites from 1 “never” to 5 “a great deal.”

There were four dependent variables (DVs): two measures of credibility perceptions—message credibility (75) and perceived source credibility (76), a persuasiveness measure adapted from Maio et al. (77) to closely fit with the topic of FH and a measure of intention to ask about the presence of allergens when eating out [adapted from Ref. (78)]. Although one might expect FH adults or parents to routinely ask about this, previous research has identified reluctance and embarrassment around asking about allergen-free food choices with a preference for written information (35). To explore the effect of concern about an FH reaction, a composite of scores for speed of reaction, reaction recency, and the recency of the most severe reaction formed a low or a high reaction salience grouping variable.^2^ Table 4 outlines the items that comprise each measure, response options, and scale reliabilities.

### Procedure

Respondents were given access to a link allowing them to complete the questionnaire, either through an email invite, social media post, or link on Allergy UK or Coeliac UK’s member websites. Respondents were initially presented with an information sheet and online consent form, outlining the study and information on participation. First, we collected basic demographic information, information about the nature and severity of the respondent’s FH, and their patterns of social media use (in general and for reasons relating to FH) followed by completion of the adapted Uses and Gratifications for Social Media measure. Second, the social validation and link-inclusion manipulations, drawing on the ELM, were then presented in one of the four randomized conditions: (1) the presence of a link and high social validation indicators, (2) no link included and high social validation, (3) link included and low social validation, and (4) no link present and low social validation. Respondents then completed the manipulation checks to ensure that they were aware of the presence of shares/likes and the inclusion or exclusion of links. Scales measuring credibility (two scales), persuasion, and intention followed these simulated tweets.^3^ The debrief information page, giving an outline of the study aims, links to further information, and the opportunity to enter a prize draw, concluded the survey. The survey took approximately 20–30 min to complete.

### Data Analysis

A principal axis factor analysis was conducted to verify our adapted 13-item Uses and Gratifications for Social Media measure. Reliability using Cronbach’s alpha values was utilized for all composite variables. Two-way multivariate analysis of variance models were conducted to explore the effect of the IVs on selected DVs. All analyses were conducted using IBM SPSS statistics software.

## Results

### Reliability of Measures

Message and perceived source credibility measures and message persuasiveness had a high level of internal consistency, as determined by Cronbach’s alpha values of >0.80 (79). The principal axis factor analysis conducted on the Uses and Gratifications for Social Media in relation to FH measure indicated that the overall Kaiser–Meyer–Olkin (KMO) verified the sampling adequacy for the analysis, KMO = 0.79 (80), and all KMO values for individual items were greater than 0.72 [acceptable limit = 0.5; (81)]. Three factors had eigenvalues over Kaiser’s criterion of 1 and in combination explained 61.12% of the variance. Following analysis of factor loadings after rotation, factor 1 represented social-seeking motivation, factor 2 represented entertainment-seeking motivation, and factor 3 information-seeking motivation. Analysis of the pattern matrix following oblique rotation highlighted that the item “I use social media in relation to food allergy/intolerance: to present myself to others as a person managing a food allergy/intolerance” loaded on both social and entertainment-seeking factors and was therefore removed. Cronbach’s alpha for the three individual gratification measures showed appropriate levels of internal consistency [all moderate to high reliability; (79)]. The measure for information-seeking bordered on the 0.70 cutoff for Cronbach’s alpha (0.67). Inclusion was justified due to the low number of measure items and negligible distance from a typical acceptance value.

### Use of Social Media Platforms

The sample of FH-concerned social media users consisted of 228 females, 21 males, and 2 respondents who did not wish to say. There were 96 parents of FH children and 155 adults with FH themselves; 76 respondents were classed as having a food allergy and 174 classified as having food intolerance (those with celiac disease and IBS-related conditions were also included in this group); one participant did not answer this question. On average, respondents used 4.24 social media platforms for general use and 2.77 platforms for reasons related to FH [difference = 1.47, 95% CI (1.25, 1.63), *t*(251) = 14.91, *p* < 0.001]. All social media platforms were used less often in relation to FH except support forums, which were used at the same frequency across both types of use. When using social media for reasons related to FH, participants unsurprisingly made more use of social media for information-seeking (M = 5.22, SD = 0.94) and social support (M = 5.20, SD = 1.17) than they did for entertainment (M = 3.69, SD = 1.30).

### Differences in Media Use for Information and Social Support

We addressed the question of whether parents (vs. FH adults) and higher (vs. lower) reaction salience groups were more motivated to use social media for information and social support with a two-way MANOVA. This indicated that there was a statistically significant main effect of being an adult or a parent on using social media for information and social support, *F*(2,241) = 3.93, *p* = 0.021, Wilks’ Λ = 0.968, partial η^2^ = 0.032. Neither the interaction effect nor the main effect of reaction salience was statistically significant. Follow-up univariate tests indicated that there was a statistically significant main effect of being an adult (M = 5.04) or a parent (M = 5.47) for using social media for social support in relation to FH issues, *F*(1, 245) = 7.66, *p* = 0.006, partial η^2^ = 0.031. There was no difference between FH adults and parents for information-seeking. These findings offer partial support for Hypothesis 1, since parents were shown to be more motivated toward using social media for social support than FH adults were, though not for information-seeking. Hypothesis 2 was not supported as reaction salience did not affect information-seeking or social support.

### The Effect of Link Inclusion and Social Validation on Twitter

Due to the specific nature and structure of Twitter, only participants who indicated that Twitter was one of the social media platforms they used (*n* = 130) were included in the analysis of responses to the Twitter stimuli. The number of participants in each condition can be seen in Table 3.

**Table 3:** Descriptives and overview for experimental manipulation conditions.

Condition	Link present	Level of social validation	*N*
1	Yes	High	29
2	No	High	35
3	Yes	Low	29
4	No	Low	37
			130

**Table 4:** Items, response options, reliability test means, and standard deviations for study measures.

Measure	Items	Response options	Reliability	Mean (SD)
Message credibility measure	How well do the following adjectives describe these Twitter posts: (1) accurate, (2) authentic, (3) believable	(1) describes very poorly to (7) describes very well	α = 0.89	4.82 (1.19)

Perceived source credibility measure	Based on your perception of the Twitter posts, please provide an evaluation in terms of the following features: (1) knowledge, (2) expertise, (3) trust, (4) reliability	(1) not very knowledgeable to (7) very knowledgeable (1) not expert to (7) expert (1) not trustworthy to (7) trustworthy (1) not reliable to (7) reliable	α = 0.96	4.54 (1.41)

Persuasiveness measure	How persuasive were the Twitter posts? Please provide your evaluation for the following questions below: (1) To what extent do you find the Twitter posts persuasive? (2) How convinced were you by the argument that asking for allergen information when eating out is a good thing? (3) To what extent were you convinced that asking for allergen information is good, specifically because it may increase the likelihood that food venues will provide the information? (4) To what extent do you agree with tweets that asking for allergen information when eating out is important?	(1) not at all to (10) extremely	α = 0.84	7.29 (1.88)

Intention measure	Please indicate how likely it is that: “If you are unsure about the presence of allergens in a dish next time you are eating out, you intend to ask for the information”	(1) unlikely to (7) likely	Single item	6.57 (0.95)

Manipulation check questions	To what extent do you feel the information posted was appreciated and shared among social media users? To what extent do these posts make use of links to other websites?	(1) Not at all to (5) Extremely (1) Never to (5) A great deal		2.97 (1.04) 3.06 (1.50)

Uses and Gratifications for Social Media	For the following section, please select to what extent you agree with each statement, beginning with the phrase—“I use social media in relation to food allergy/intolerance”: *Information-seeking motivation* (1) … so that I don’t miss the important issues of the day (2) … to know others’ opinions about food allergy/intolerance (3) … to understand a range of views relating to food allergy/intolerance (4) … to get useful information relating to food allergy/intolerance	(1) strongly disagree to (7) strongly agree	α = 0.67	5.22 (0.94)
	*Entertainment-seeking motivation* (5) … because it is fun (6) … because I enjoy it (7) … to relieve boredom (8) … to relax		α = 0.86	3.69 (1.30)
	*Social-seeking motivation* (9) … to connect with other users that are concerned with food allergy/intolerance (10) … to get support from other people with food allergies/intolerances (11) … to feel like I belong to a community of food allergic/intolerant people (12) … to talk about food allergy/intolerance with others		α = 0.85	5.20 (1.17)

#### Manipulation Checks

An independent samples *t*-test showed that participants in the high likes/shares condition were more likely to indicate that the tweets were appreciated by others (M = 3.23) than those in the low likes/retweets condition (M = 2.72) *t*(132) = 2.90, *p* = 0.004. A second analysis indicated that participants in the links-included condition were more likely to report that the tweets make use of links (M = 4.28) than those in the no-links-included condition (M = 2.07) *t*(122.125) = 13.265, *p* < 0.001. In sum, both manipulations were successful.

#### Main Analysis

A two-way MANOVA was run with two IVs—link presence and social validation level—and four DVs—message credibility, source credibility, persuasion, and intention. To control for the possible effect of information-seeking and social-support orientations, these variables were added as covariates. Hypothesis 3 was not supported as there was no statistically significant main effect of social validation level on the DVs. There was no interaction effect. In support of Hypothesis 4, there was a small but statistically significant main effect of link presence on the DVs, *F*(4, 121) = 3.78, *p* = 0.006, Wilks’ Λ = 0.89, partial η^2^ = 0.11.

Follow-up of the significant main effect of link presence with univariate two-way ANOVAs demonstrated that there was a statistically significant main effect of link presence (M = 5.17) vs. link exclusion (M = 4.54) for message credibility—*F*(1, 124) = 10.97, *p* = 0.001, partial η^2^ = 0.08. There was also a main effect of link presence on persuasion (link presence M = 7.64, link omission M = 7.01)—*F*(1, 124) = 5.68, *p* = 0.019, partial η^2^ = 0.04. There were no significant differences for source credibility and intention. Thus, Hypothesis 4 was partially supported since the inclusion of a link in a tweet enhanced perceptions of source credibility and persuasiveness.

## Discussion

This study first investigated the way in which FH-concerned individuals used social media, noting that both adults and parents utilized platforms primarily for information and social reasons. However, parents of FH children used social media for social support significantly more than FH adults. The study also explored how socially validated information and inclusion of links in tweets affected inferences about the credibility of tweets, persuasiveness of the tweet content, and intention to act upon the information. There was no effect of social validation (number of likes/retweets) but the inclusion of a link increased perceived credibility of the message and persuasiveness of tweet content.

Previous research has suggested that Twitter is primarily used for either information or social purposes (53, 54). Our findings support UGT research around social media use [e.g., Ref. (51–54)], demonstrating that users are interested in FH, either for their own food sensitivity or their child’s, value social media platforms for seeking social support, but also for information provision. However, although there were no differences in relation to using social media for information provision, parents of children with allergies/intolerances were more strongly oriented to use social media for social-support reasons than were adult sufferers. This is in line with Broome et al.’s (57) findings around an FH parent’s need to develop a sense of expertise in FH through the use of the Internet and online parenting communities, seeking the knowledge of other parents with FH children. It also links with Begen et al.’s (55) and Cummings et al.’s (56) observations around concerns and challenges associated with the care of FH children specifically, which are eased through support and advice of others in similar parenting circumstances—as noted by Broome et al. (57). A higher salience of a potential FH reaction was not associated with a greater use of information or social support on social media. It may be the case that having any form of negative reaction to a food allergen is enough to promote a desire to seek out information and support relating to one’s condition. It may also be that it is the day-to-day routines of needing to eat, buy, and prepare dishes without the problematic allergen(s) that are the trigger for information-seeking and social support rather than the severity and recency of previous reactions.

Responses to the experimental manipulation demonstrated that the presence of links in Twitter posts had a positive effect on ratings of message credibility, as well as of persuasiveness but not on ratings of source credibility or intention. The level of social validation for each Twitter post did not alter user perceptions of any of these measures. The findings of Park et al. (82) on the effects of product reviews suggest that the inclusion of the links represented a cue to quality, a validation of the content, thus increasing the credibility and persuasiveness of the tweets for the invested FH users in our sample. The ELM might suggest that our sample of FH-concerned users would be more likely to carefully consider (centrally process) the tweet content (textual information within the tweet), rather than attending to the more peripheral cues provided by the likes and retweets. Further in line with ELM, a more knowledgeable and involved audience will favor a central processing route, as they are more motivated to attend to and understand the message content (83). The effect of link inclusion on message credibility but not *source* credibility specifically may further reflect a preference toward central processing; the peripheral position of the tweet-author in the experimental stimuli may have meant that participants were not paying attention to the source (tweet-author) at all. Contrary to the evidence that in reality FH-concerned individuals are restrained in asking about allergens when eating out [e.g., Ref. (35)], the overall study mean for intention here suggests a high willingness to ask. This may show a ceiling effect, but may also reflect a more engaged audience (i.e., a volunteering sample).

The lack of effect of social validation (likes and retweets) manipulation is perhaps more surprising, given the high value participants attributed to social media for providing social support around FH and the routine use of rules of thumb for making judgments about allergy management (73). It may also be that in the unfamiliar study context, participants did not rely on these rules of thumb but rather preferred to bypass the more peripheral cues and scrutinize the content of the arguments closely in order to decide whether or not to trust it (83)—particularly given how important it is for those with FH to make good decisions about the presence of allergens in food (35, 73). The absence of an effect of retweets and likes is also in line with the work by Waddell (66). He considers the notion that, contrary to the assumption we have made, such features may not be considered as social validation but rather as statistical information that is difficult to process (67). However, the Waddell study also considered the effect of a richer set of cues in terms of comment valence—one might expect that more minimal indicators of social validation may be less likely to have an effect in this context.

Our research further progresses our understanding of the affordances of social media [e.g., (1)]. We have seen that those who are utilizing platforms for information or social reasons, or around a health topic, are likely to be influenced by the presence of external websites/links. Linking to additional evidence is likely to increase perceptions of credibility and the persuasiveness of the information. Moreover, a greater understanding of the features of Twitter posting specifically in relation to food issues furthers our understanding of how to approach managing risk communication more appropriately around a topic like FH, for example, during times of emergency (23). Support organizations and public health bodies would do well to integrate the use of links into their social media policies and encourage users to click links for further details. In line with Miller and Bell (22), this would assist users (especially those who are less experienced with social media use) in distinguishing more trustworthy information online. By the same token, however, it is equally possible that unofficial advice about allergy may be considered as more credible and persuasive simply by virtue of containing a link to other sources. Such concerns were raised in discussions around the issue of evaluating the quality and inaccuracies of online information by Flanagin and Metzger (62), and Lee et al. (63).

### Limitations and Future Research

Participants in this study are by definition those with a particular interest and involvement in the issue of FH. It would be beneficial to explore effects with a broader sample—or indeed include measures to characterize the exact degree of involvement and interest in the issue under scrutiny. This would give greater clarity as to whether the effect of the presence of links remains with a less-invested sample, and/or if the level of social validation has an effect on perceptions of credibility (through a peripheral processing route). It would also be useful to further disentangle the effect of links (e.g., tiny URLs/shortened links, links that do not state the source in the web-address, etc.). In fact, it is impossible to separate the effect of inclusion of links to web-addresses like the Food Standards Agency, BBC news, and Anaphylaxis Campaign (a leading allergy charity in the UK) or the presence of links generally on subsequent ratings. In addition, a study design that allowed participants to access a link and included this as either an IV or a DV would be highly informative. As well as broadening or better characterizing the participant group and extending the manipulations, there is also the more basic issue that may undermine the results of the present study, that is, whether retweets and favorites actually convey social validation. One of the ways in retweets can be used is to “call out” the author of the tweet and to add a comment that expresses a different view. It might therefore be useful to establish whether retweets and favorites may be viewed differently, vary in the extent to which they represent social validation, and thus should be disaggregated.

A further limitation of the present study is that it provides no understanding of the extent to which the effects we have seen (and not seen) are a function of the sources (authors) of the tweets. There is a long history of offline research looking at the effects of source credibility (84, 85), and it is quite possible that the effect of link inclusion would be mediated by the way in which the source/author is regarded. This in turn is likely to be a function of the person viewing the source. It might be, for example, that parents would likely see other parents’ accounts on Twitter or Facebook as more credible than those with FH themselves would.

### Conclusion

Social media use around FH is valued both for the information and for the social support that it provides. The inclusion of links within tweets increased ratings of message credibility and persuasiveness of the post-content. This, and the lack of impact of social validation indicators such as retweets and favorites, appears to indicate that in the domain of a health issue, such as FH, that in the online setting of Twitter, information is centrally processed. Consequently, links are potentially a valuable asset for health-concerned users to attend to useful or essential information *via* social media. The concerned health community of those with FH valued the information within posts rather that the cues provided as to the popularity of the post. In support of Coulson (32), it is crucial to understand the affordances of the different social media platforms, in order to know how to better support online communities who use them to help manage health conditions.

## Ethics Statement

This study was carried out in accordance with the recommendations of the British Psychological Society Code of Ethics and Conduct with informed consent from all respondents. All respondents gave informed consent in accordance with valid consent procedures in line with Internet-mediated research participation. The protocol was approved by the Department of Psychology’s ethics committee at the University of Bath (reference number: 16-275).

## Author Contributions

RH designed and conducted the reported study and produced the written manuscript. JB assisted in the study design and provided detailed comment and amendments to various manuscript versions. JL assisted in the study planning and provided a detailed comment on the various versions of the manuscript.

## Conflict of Interest Statement

The authors declare that the research was conducted in the absence of any commercial or financial relationships that could be construed as a potential conflict of interest.

## Footnotes

^1^https://www.qualtrics.com/ (Accessed: March 2, 2018).

^2^Further details on the structure and creation of this composite variable can be requested from the corresponding author for this paper.

^3^To provide information for another study, respondents were finally asked if they could identify any social media users that they considered to be expert or trustworthy. This information is not reported here.
